# Proteomic Analysis of a Novel *Bacillus* Jumbo Phage Revealing Glycoside Hydrolase As Structural Component

**DOI:** 10.3389/fmicb.2016.00745

**Published:** 2016-05-18

**Authors:** Yihui Yuan, Meiying Gao

**Affiliations:** Key Laboratory of Agricultural and Environmental Microbiology, Wuhan Institute of Virology, Chinese Academy of SciencesWuhan, China

**Keywords:** jumbo bacteriophage, structural proteome, glycoside hydrolase

## Abstract

Tailed phages with genomes of larger than 200 kbp are classified as Jumbo phages and exhibited extremely high uncharted diversity. The genomic annotation of Jumbo phage is often disappointing because most of the predicted proteins, including structural proteins, failed to make good hits to the sequences in the databases. In this study, 23 proteins of a novel *Bacillus* Jumbo phage, vB_BpuM_BpSp, were identified as phage structural proteins by the structural proteome analysis, including 14 proteins of unknown function, 5 proteins with predicted function as structural proteins, a glycoside hydrolase, a Holliday junction resolvase, a RNA-polymerase β-subunit, and a host-coding portal protein, which might be hijacked from the host strain during phage virion assembly. The glycoside hydrolase (Gp255) was identified as phage virion component and was found to interact with the phage baseplate protein. Gp255 shows specific lytic activity against the phage host strain GR8 and has high temperature tolerance. *In situ* peptidoglycan-hydrolyzing activities analysis revealed that the expressed Gp255 and phage structural proteome exhibited glycoside hydrolysis activity against the tested GR8 cell extracts. This study identified the first functional individual structural glycoside hydrolase in phage virion. The presence of activated glycoside hydrolase in phage virions might facilitate the injection of the phage genome during infection by forming pores on the bacterial cell wall.

## Introduction

Bacteriophages are viruses infecting bacteria and exhibit extremely high abundant and diversity (Rohwer, 2003; Mizuno et al., 2013). Tailed phages with genomes larger than 200 kbp are known as “Jumbo phages” or “giant phages” (Hendrix, 2009). Although the Jumbo phage was discovered 47 years ago (*Bacillus* phage G), they have rarely been isolated, and so far, only the genomes of 82 Jumbo phages have been deposited in the GenBank database. The newly isolated Jumbo phages usually exhibit low genome similarity to the previously isolated ones and contain numerous genes of unknown function (Lecoutere et al., 2009; Abbasifar et al., 2014). Hence, genomic annotation of Jumbo phages is often disappointing. Structural proteome analysis of phages has been proved to an effective approach for identifying unknown phage structural proteins and for discovering new phage structural proteins. Proteomic analysis of *Pseudomonas* Jumbo phage 201ϕ2-1 virions revealed that the RNA polymerase β or β′ subunit is component of phage virions and this phage contains more structural proteins than the smaller phages (Thomas et al., 2008, 2010). In our lab, a novel *Bacillus pumilus* Jumbo phage, vB_BpuM_BpSp, was isolated and sequenced. The phage exhibits extremely low genome similarity to the existing biological entities and its virions contain several unique substructures, including the curly tail fibers (unpublished data). Genomic annotation of phage vB_BpuM_BpSp (GenBank accession number KT895374) identified eight phage structural proteins, including four proteins with no exact function assigned. In this study, we analyzed the structural proteome of the Jumbo phage vB_BpuM_BpSp, and our results show that an individual glycoside hydrolase is component of the phage virion and it was found to interact with the baseplate protein. The existence of a glycoside hydrolase as phage structural component might facilitate the infection of phage vB_BpuM_BpSp to the host strain *B. pumilus.*

Glycoside hydrolases are encoded by microbes and plants to catalyze the hydrolysis of polysaccharides (Bissaro et al., 2015). The function of glycoside hydrolase is similar to that of phage endolysin, which degrades the bacterial cell wall and facilitate the release of mature progeny phages (Hermoso et al., 2007). Several phages employ cell wall hydrolases to facilitate infection. The tail fiber protein of *Escherichia coli* phage T5 acts as a peptidoglycan hydrolase to form pores on the bacterial cell wall to transfer phage genome DNA (Boulanger et al., 2008). The tail spike proteins of *Salmonella* phage P22, *Shigella* phage Sf6, *E. coli* phage HK620 and K5A all exhibit glycoside hydrolase activity (Chen and King, 1991; Bhardwaj et al., 2011). Phage Basilisk contains a chitinase as a virion structural component. However, the function of the chitinase is unknown (Grose et al., 2014). The existence of the glycoside hydrolase domains on phage tail fiber and tail spike proteins could facilitate host recognition and infection of phages, through the binding and degradation of host lipopolysaccharides (Thompson et al., 2010). Genes encoding glycoside hydrolases have been found in several phage genomes (Maaroufi and Levesque, 2015). These discovered glycoside hydrolase domains all are the part of phage structural proteins, and to our knowledge, no individual glycoside hydrolase that might benefit the phage infection has been found. Comparison to phages with small genomes, the Jumbo phages contain large dsDNA genomes. Thus, the injection of Jumbo phage genomes is much more difficult and time consumption. To date, no mechanism that facilitates Jumbo phage genome injection and phage infection has been reported.

## Materials and methods

### Bacterial strains and growth conditions

Strains and plasmids used in this study are shown in Table 1. All strains were cultured in LB broth with moderate shaking.*E. coli* M15 and BL-21 were used for protein expression. *Bacillus anthracis* CMCC63605, *Bacillus thuringiensis* HD-73, *Bacillus cereus* 411A, *Bacillus subtilis* 168, *Pseudomonas aeruginosa* PAO1 (ATCC47085), *Yersinia pseudotuberculosis* NaI were collected by our lab and used for the lytic spectrum test. Antibiotics were used at a concentration of 100 μg/ml for ampicillin and 34 μg/ml for kanamycin.

**Table 1:** **Strains and plasmids used in this study**.

**Bacteria, plasmid, or primer**	**Relevant characteristics**	**Reference or resource**
**BACTERIA**		
***B. pumilus*** **strains**		
GR8	*B. pumilus* strain, pathogen of ginger rhizome rot disease, host strain of phage vB_BpuM_BpSp	Peng et al., 2013
***E. coli*** **strain**		
BL21(DE3)	F^−^*dcm ompT hsd*S(${\text{r}}_{\text{B}}^{-}$${\text{m}}_{\text{B}}^{-}$)*gal* λ(DE3)	Novagen
M15	*lac gal mtl rec*A^+^ *uvr*^+^ [pREP4 *lac*I Kan^R^]	Qiagen
**PLASMIDS**		
pET-28a(+)	Expression vector; Kan^r^, C/N-terminal His tag/thrombin/T7 tag, T_7_ *lac* promoter, T7 transcription start, f1 origin, *lacI*	Novagen
pET/*gp255*	Kan^r^, *gp*255 of phage vB_BpuM_BpSp was cloned into pET-28a(+) *Bam*H I/*Sal* I site	This study
pET/*gp255C*	Kan^r^, 3′ termini 642 bp of *gp*255 of phage vB_BpuM_BpSp was cloned into pET-28a(+) *Bam*H I/*Sal* I site	This study
pET/*gp255N*	Kan^r^, 5′ termini 567 bp of *gp*255 of phage vB_BpuM_BpSp was cloned into pET-28a(+) *Bam*H I/*Sal* I site	This study
pQE30	Expression vector; Amp^r^, N-terminal His tag, T_5_ *lac* promoter, T5 transcription start, ColE1 origin, *lacI*	Qiagen
pQE/*gp287*	Amp^r^, *gp*287 of phage vB_BpuM_BpSp was cloned into pQE30 BamH I/*Sal* I site	This study
**PRIMERS**		
*gp*255-F/*Bam*H I	GAGGATCCAAAGGTACAGTTGACG, forward primer to amplify *gp255*	This study
*gp*255-R/*Sal* I	GCGTCGACAATACCATTAATTAGAC, reward primer to amplify *gp255*	This study
*gp*255-R567/*Sal* I	GCGTCGACAATACCATTAATTAGAC, reward primer to amplify *gp255N*	This study
*gp*255-F568/*Bam*H I	GAGGATCCAAAGGTACAGTTGACG, forward primer to amplify *gp255C*	This study
*gp*287-F/*Bam*H I	GAGGATCCATGAATGATTTTAAGG, forward primer to amplify *gp287*	This study
*gp*287-F/*Sal* I	CTGTCGACCTATAAATATATTTTTG, reward primer to amplify *gp287*	This study

### Structural proteome analysis of phage virions

Phages purified by sucrose density gradient centrifugation were used for SDS-PAGE (Sodium Dodecyl Sulfate Polyacrylamide Gel Electrophoresis) analysis as described before (Yuan et al., 2015). After separation by 15% SDS-PAGE, the gel was stained b with Coomassie blue and destained with water. The proteins used for HPLC-ESI-MS/MS (High Performance Liquid Chromatography Electrospray Tandem Mass Spectrometry) analysis were separated by 15% SDS-PAGE for 20 min, and the 1.5-cm gel region containing partially separated proteins was excised, followed by in-gel digestion and MS analysis. HPLC-ESI-MS/MS was performed on a linear ion trap mass spectrometer LTQ Orbitrap Velos (Thermo Fisher, Massachusetts, USA). The MS data was analyzed using Mascot version 2.3.0 and Sequest 1.2.0.208 against the local database of all possible phage proteins and host strain proteins, and the online NR (non-redundant protein sequence) database in NCBI.

### Bioinformatic analysis of protein Gp255

The functional domain composition of protein Gp255 was predicted using Pfam database (Punta et al., 2012), Conseved Domain Database (Marchler-Bauer et al., 2013), PDB database (http://www.rcsb.org/pdb/home/home.do), and HHpred (Soding et al., 2005). The phage proteins belonging to glycoside hydrolase family 25 (GH25) were found by searching the CAZy database (http://www.cazy.org/GH25_viruses.html; Henrissat and Bairoch, 1996). Amino acid sequences of Gp255 and similar proteins were aligned using ClustalW 2.0 (Larkin et al., 2007) and Mega 6.0 (Tamura et al., 2013).

### Expression, purification, and functional analysis of *gp*255 and *gp*287

DNA manipulation was carried out as described before (Sambrook et al., 2001). The primers used and plasmids constructed in this study are shown in Table 1. Gene *gp*255 and the truncated *gp*255 were amplified from the phage genome and inserted into the *Bam*H I/*Sal* I sites of plasmid pET28a. The recombinant plasmid was transformed into *E. coli* BL21 to generate recombinant *E. coli* strain for protein expression. The proteins was expressed by induction with 0.4 mM IPTG (isopropyl-β-D-thiogalactopyranoside) and purified by Ni-NTA (nickel-nitrilotriacetic acid) columns (Qiagen, Dusseldorf, Germany) and buffer exchanged into 50 mM Tris (pH 8.0), 300 mM NaCl, 10% glycerol, and 1 mM β-mercaptoethanol. The lytic activities of Gp255 against *B. pumilus* GR8 and other strains used for lytic spectrum assay were investigated as described previously (Yuan et al., 2012). The exponential-growth cells were collected and resuspended in the reaction buffer (50 mM Tris [pH8.0], 300 mM NaCl, 10% [vol/vol] glycerol, and 1 mM β-mercaptoethanol) at about 0.8 optical density at 600 nm. Purified protein Gp255 was added into the bacterial suspension to a final concentration of 0.05 μM and the absorbance of the bacterial suspension at 600 nm was tested at an interval of 30 s at 37°C. Protein Gp255 used for Far western blotting analysis was labeled with biotin as the manufactures' instruction (Roche, Basel, Switzerland).

The gene *gp*287 was amplified from the phage genome by using primer pairs *gp*287-F/*Bam*H I and *gp*287-R/*Sal* I, and inserted into the *Bam*H I/*Sal* I sites of plasmid pQE30. The recombinant plasmid was transformed into *E. coli* M15 to produce recombinant *E. coli* strain. Protein Gp287 was expressed by inducing the strain with 0.4 mM IPTG and purified by Ni-NTA columns.

### Analysis of the peptidoglycan-hydrolyzing activities of the phage vB_BpuM_BpSp virion proteome

The peptidoglycan-hydrolyzing activities of the structural proteomic proteins of phage vB_BpuM_BpSp were analyzed by zymogram analysis as described previously (Buist et al., 1995). The exponential growth bacterial strains were collected by centrifugation and sterilized at 121°C for 20 min before use. The phage proteomic proteins and Gp255 were separated using 12% SDS-PAGE containing 0.4% autoclaved GR8 cells. After separation, the gel was rinsed with 2.5% Triton X-100 for four times (20 min each) at room temperature and was incubated overnight at 37°C in renaturation buffer (50 mM 2-morpholinoethanesulfonic acid, 1% Triton X-100, 1 mM MgCl_2_, 1 mM CaCl_2_, pH 6.5). After that, the gel was rinsed with distilled water and stained with 1% methylene blue in 0.01% KOH for 2 h, and subsequently destained with distilled water.

### Identification of proteins that interact with Gp255

To identify the phage structural proteins that intact with Gp255, purified phage virions were separated by 15% SDS-PAGE and transferred onto PVDF membrane (Millpore, Massachusetts, USA). Far western blotting was carried out as described previously (Javed et al., 2015) by using the biotin-labeled Gp255 at a hybrid concentration of 5 μg/ml. The protein band in the gel corresponding the hybridization band on the PVDF membrane was sliced and in-gel digested, followed by identification with MALDI-TOF-TOF MS (Matrix-assisted Laser Desorption Ionization Tandem Time of Flight Mass Spectrometry) on an Ultraflex MALDI-TOF/-TOF mass spectrometer (Bruker, Karlsruhe, Germany; Lavigne et al., 2006). To verify the interaction of Gp255 with the identified protein Gp287, purified Gp287 was separated by SDS-PAGE, and Far western blotting was carried out as described above. The Western blotting of Gp287 was performed by using the mouse monoclonal antibody anti-His_6_ (Roche, Basel, Switzerland), and biotin-labeled Gp255.

## Results

### Structural proteome of jumbo phage vB_BpuM_BpSp

#### General features of jumbo phage vB_BpuM_BpSp structural proteome

**Figure 1:**
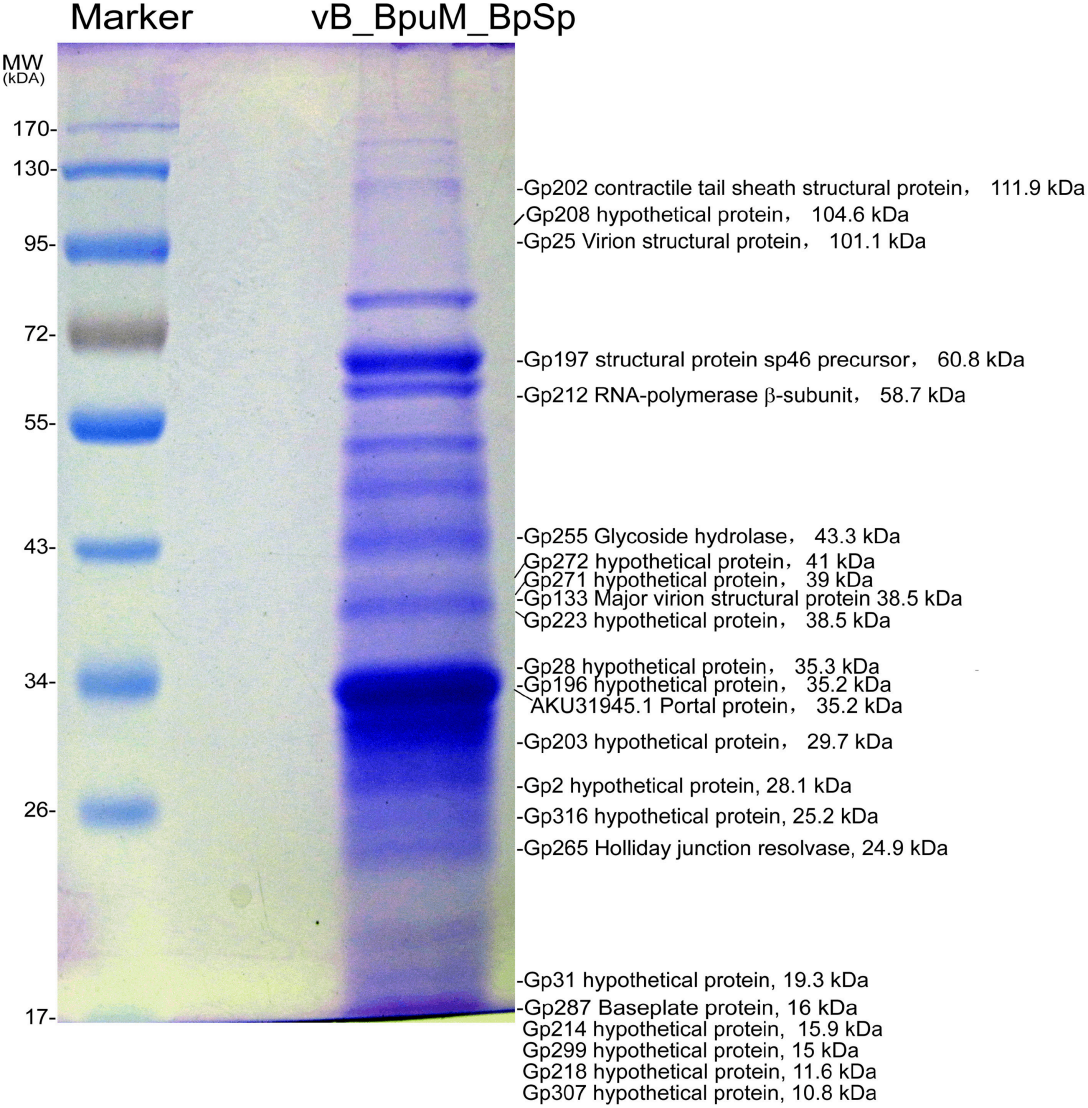
**Proteomic analysis of the structural proteins of phage vB_BpuM_BpSp**. The phage purified by sucrose density gradient centrifugation was analyzed by SDS-PAGE and the protein bands were further identified by HPLC-MS/MS. The molecular weight standard is indicated on the left. The corresponding CDSs, the predicted functions, and the predicted molecular mass of the proteins identified by mass spectrometry were also indicated.

Genomic analysis revealed that eight genes in Jumbo phage vB_BpuM_BpSp genome predictably code phage structural proteins, including virion structural protein (Gp25), major virion structural protein (Gp133), prohead core scaffold protein and protease (Gp191), structural protein precursor (Gp197), tail sheath protein (Gp202), portal vertex protein (Gp204), virion structural protein (Gp269), and baseplate protein (Gp287), whereas the genes encoding the phage capsid protein, tail tube protein, and tail fiber protein, are unknown. To identify the structural proteins in the mature phage virion of vB_BpuM_BpSp, purified phage virions were analyzed using SDS-PAGE followed by HPLC-MS/MS. In total, 23 structural proteins were identified (Table 2; Figure 1), including 22 phage-coding proteins and one host bacteria-coding protein. Among the 22 phage-coding proteins, 14 of them showed unknown functions, five were annotated as phage structural protein (virion structural protein, major virion structural protein, structural protein precursor, contractile tail sheath protein, and baseplate protein), and the other three proteins are glycoside hydrolase, Holliday junction resolvase, and RNA polymerase (RNAP) β subunit. In addition, by searching the MS data against the genome of the host strain *B. pumilus* GR8, one protein band was identified as phage portal protein (GenBank accession number AKU29927.1). Although three proteins, Gp191, Gp204, and Gp269, were annotated as phage structural proteins by genomic analysis, the corresponding bands were not identified on the SDS-PAGE. This might be due to the low copy numbers of these proteins in phage virion.

**Table 2:** **Characteristic of the vB_BpuM_BpSp virion proteome identified by HPLC-ESI_MS/MS**.

**Gp**	**Molecular mass (kDa)**	**No. of identified peptides**	**Coverage (%)**	**Predicted function**
Gp2	28.1	2	6.3	Hypothetical protein
Gp25	101.1	8	9.3	Virion structural protein
Gp28	35.3	5	17.4	Hypothetical protein
Gp31	19.3	4	4.2	Hypothetical protein
Gp133	38.5	5	17.7	Major virion structural protein
Gp196	35.2	5	14.2	Hypothetical protein
Gp197	60.8	8	15.2	Structural protein precursor
Gp202	111.9	13	15	Contractile tail sheath protein
Gp203	29.7	6	27.5	Hypothetical protein
Gp208	104.6	8	9.3	Hypothetical protein
Gp212	58.7	6	13.9	RNA polymerase β-subunit
Gp214	15.9	3	19.6	Hypothetical protein
Gp218	11.6	3	29.6	Hypothetical protein
Gp223	38.5	3	11.6	Hypothetical protein
Gp255	43.3	6	14.9	Glycoside hydrolase
Gp265	24.9	2	8.6	Holliday junction resolvase
Gp271	39	6	26.2	Hypothetical protein
Gp272	41	11	31.3	Hypothetical protein
Gp287	16	2	30.9	Baseplate protein
Gp299	15	5	23.5	Hypothetical protein
Gp307	10.8	2	12.2	Hypothetical protein
Gp316	25.2	2	10.3	Hypothetical protein
AKU31945.1^a^	34.2	5	19.2	Phage portal protein

a GenBank accession number of the proteins from host strain GR8.

According to the bioinformatic analysis of the Jumbo phage vB_BpuM_BpSp genome, the genes encoding these 22 identified phage-coding structural proteins are mainly located at three separate regions in the genome, except for gene *gp*133, which is far from the three gene subclusters. The first gene cluster contains *gp*25, *gp*28, and *gp*31, whose functions are unknown. The second gene cluster contains nine genes from *gp*196 to *gp*223; the functions of most of them (*gp*196, *gp*203, *gp*208, *gp*214, *gp*218, and *gp*223) are unknown, except the following: structural proteins precursor gene (*gp*197), contractile tail sheath protein gene (*gp*202), and RNA polymerase (RNAP) βsubunit gene (*gp*212). The third gene cluster is composed of nine genes from *gp*255 to *gp*316, including the genes for glycoside hydrolase (*gp*255), Holliday junction resolvase (*gp*265), baseplate protein (*gp*287), and other genes with unknown functions (*gp*271, *gp*272, *gp*299, *gp*307, *gp*316, and *gp*2).

#### Candidate genes for phage curly tail fibers

A notable feature of the Jumbo phage vB_BpuM_BpSp is their curly tail fibers. The curly tail fibers have also been observed on the virions of *B. thuringiensis* phage 0305ϕ8-36, and the proteins ORF119, ORF129, and ORF131 of phage 0305ϕ8-36 have been speculated to be candidate curly tail fiber proteins (Thomas et al., 2007). However, such candidates for other phages remain unknown. Comparative proteomic analysis of the phages, vB_BpuM_BpSp and 0305ϕ8-36, revealed that two of the identified structural proteins (Gp25 and Gp133) of the phage vB_BpuM_BpSp are similar to the proteins, ORF154 and ORF129, of phage 0305ϕ8-36, respectively. Protein ORF154 was also identified as the associated tail protein in phage 0305ϕ8-36, although its exact function is unknown. Though the phages, vB_BpuM_BpSp and 0305ϕ8-36, exhibit extremely low similarity in genome sequences, they have similar tail fiber structures. The homologous structural protein coding genes, *gp*25 and *gp*133, might be candidate genes for curly tail fiber protein.

#### Candidate genes for phage capsid protein

Previous analysis of phage virion proteomes has revealed that the major capsid protein and tail sheath protein are the most abundant proteins in phage virions (Miller et al., 2003; Lecoutere et al., 2009; Chan et al., 2014). Genome annotation of the phage vB_BpuM_BpSp revealed that its capsid proteins are unknown. In this study, the most abundant protein bands on the SDS-PAGE were identified to that of Gp196 and Gp197 (Figure 1). By analyzing these two proteins using HHpred, we found that Gp197 contains head vertex protein domain, whereas no functional domain was found in protein Gp196. Among the genes near *gp*196 and *gp*197 in the phage vB_BpuM_BpSp genome, *gp*191, *gp*204, and *gp*198 encode prohead core scaffold protease, portal vertex protein, and terminase large subunit, respectively. Genes with related functions usually form gene clusters in phage genomes (Thomas et al., 2007). Considering the location of these two genes in the phage genome and the results of their bioinformatic, we postulate that *gp*197 might be the major phage capsid protein gene.

### Bioinformatic features of Gp255

**Figure 2:**
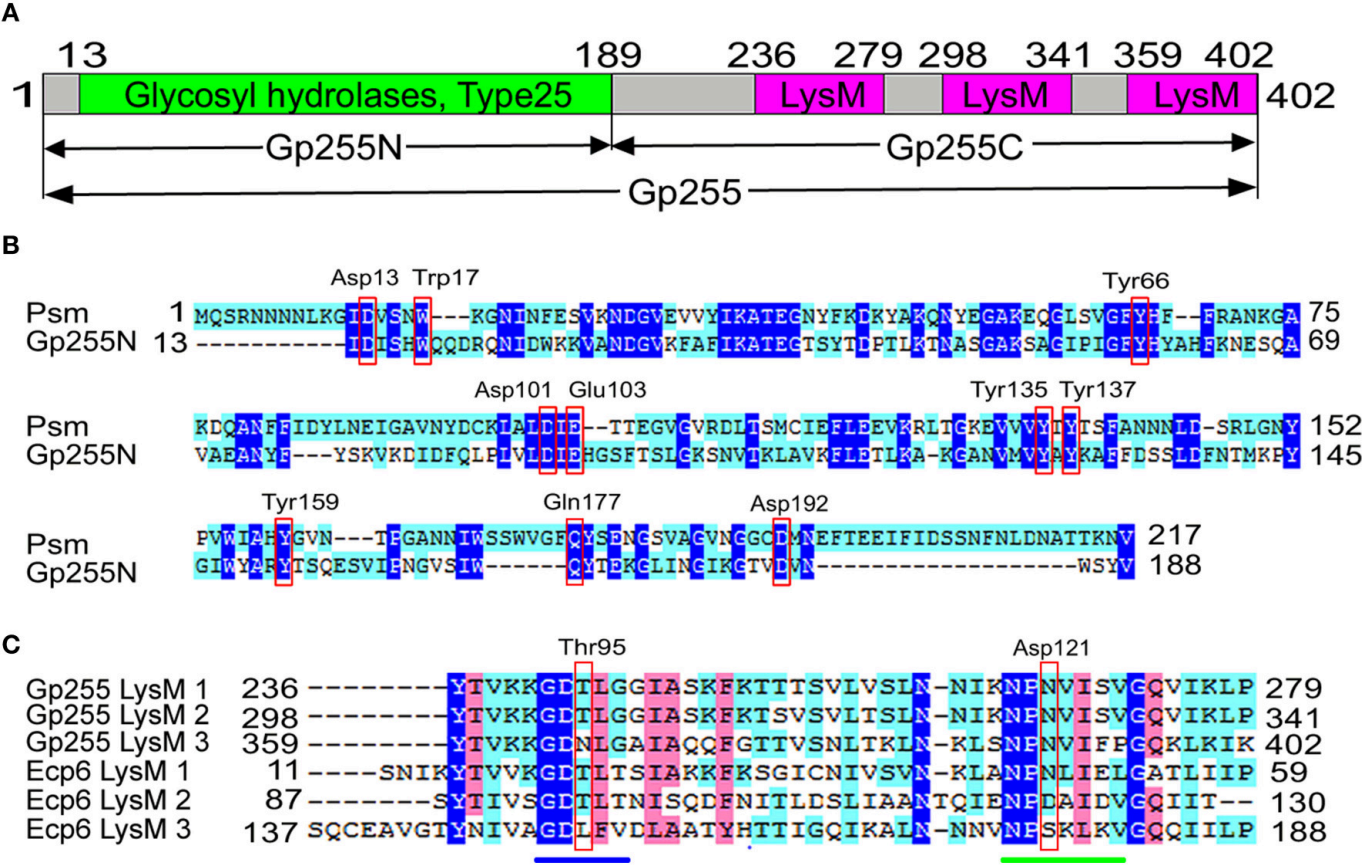
**Schematic diagram of Gp255 and amino acid sequence alignment of the LysM domains. (A)** The domain composition of Gp255 analyzed by using Pfam and the positions of the amino acid residues at the end of each domain are indicated. The N-terminal catalytic domain and C-terminal cell wall binding domains are shown. **(B)** Alignment of the catalytic domain of Gp255 and endolysin Psm (PDB database accession number 4KRU_A) from phage phiSM101. The catalytic site residues of endolysin Psm are indicated in red box. **(C)** Alignment of the amino acid sequence of the three LysM domains from Gp255 and the three LysM domain from fungal efector Ecp6 (PDB database accession number 4B8V_A). The positions of the three peptides in Gp255 and Ecp6 are indicated. The two chitin-binding sites in LysM1 and LysM3 of Ecp6 are indicated with a blue line and a green line. The chitin-binding sites of LysM2 of Ecp6 are indicated in red box. The sequences were aligned by ClustalW 2.0.

Structural proteomic analysis of phage vB_BpuM_BpSp virion revealed that six peptides were mapped to the glycoside hydrolase (Gp255), suggesting Gp255 is the structural component of the phage virion and it is located in the third gene subcluster of the structural protein-associated genes in the phage genome (Figure 1). The G+C content of gene *gp*255 is 30.27%, which is a little higher than that of the phage genome (25.9%). Structural analysis of the proteins indicates that protein Gp255 contains a N-terminal glycosyl hydrolase catalytic domain, with four of the six peptides identified by MS mapped to this domain, and three LysM peptidoglycan binding domains, with one peptide identified by MS mapped to the first LysM domain (Figure 2A). Though only low similarity was observed, the catalytic domain contain conserved catalytic site residues as the endolysin of *Clostridium perfringens* phage phiSM101 (Figure 2B) (Tamai et al., 2014). All three LysM domains have 43 amino acid residues and the first one differs from the second one by only three amino acid residues, whereas the third one shows low similarity to the first and the second ones (Figure 2C). Compared to the LysM domains of fungal effector Ecp6, the three LysM domains of Gp255 contain conserved chitin-binding sites for peptidoglycan binding (Sánchez-Vallet et al., 2013). A BlastP search of the protein in the NR database in NCBI revealed that Gp255 exhibits only 23.7% similarity to the endolysin of the *Lactobacillus fermentum* phage ϕPYB5 (Wang et al., 2008), but the protein has higher similarity to bacterial glycoside hydrolases. Based on the similarities of Gp255 to other glycosyl hydrolases, Gp255 belongs to the glycoside hydrolase family 25 (GH25). Among the glycoside hydrolase of GH25 family, 128 proteins encoded by the phage genome were found in the CAZy database and these protein have been annotated largely as phage endolysins. Other than Gp255, there are two more genes that encode polysaccharide hydrolases in the phage vB_BpuM_BpSp genome. These two polysaccharide hydrolases are N-acetylmuramoyl-L-alanine amidase (Gp019) and spore cortex-lytic enzyme (Gp067). The N-acetylmuramoyl-L-alanine amidase could be the phage endolysin that lyse the host cell to release mature progeny phages, while the function of spore cortex-lytic enzyme to phage vB_BpuM_BpSp is unknown.

### Functional characterization of glycoside hydrolase Gp255

**Figure 3:**
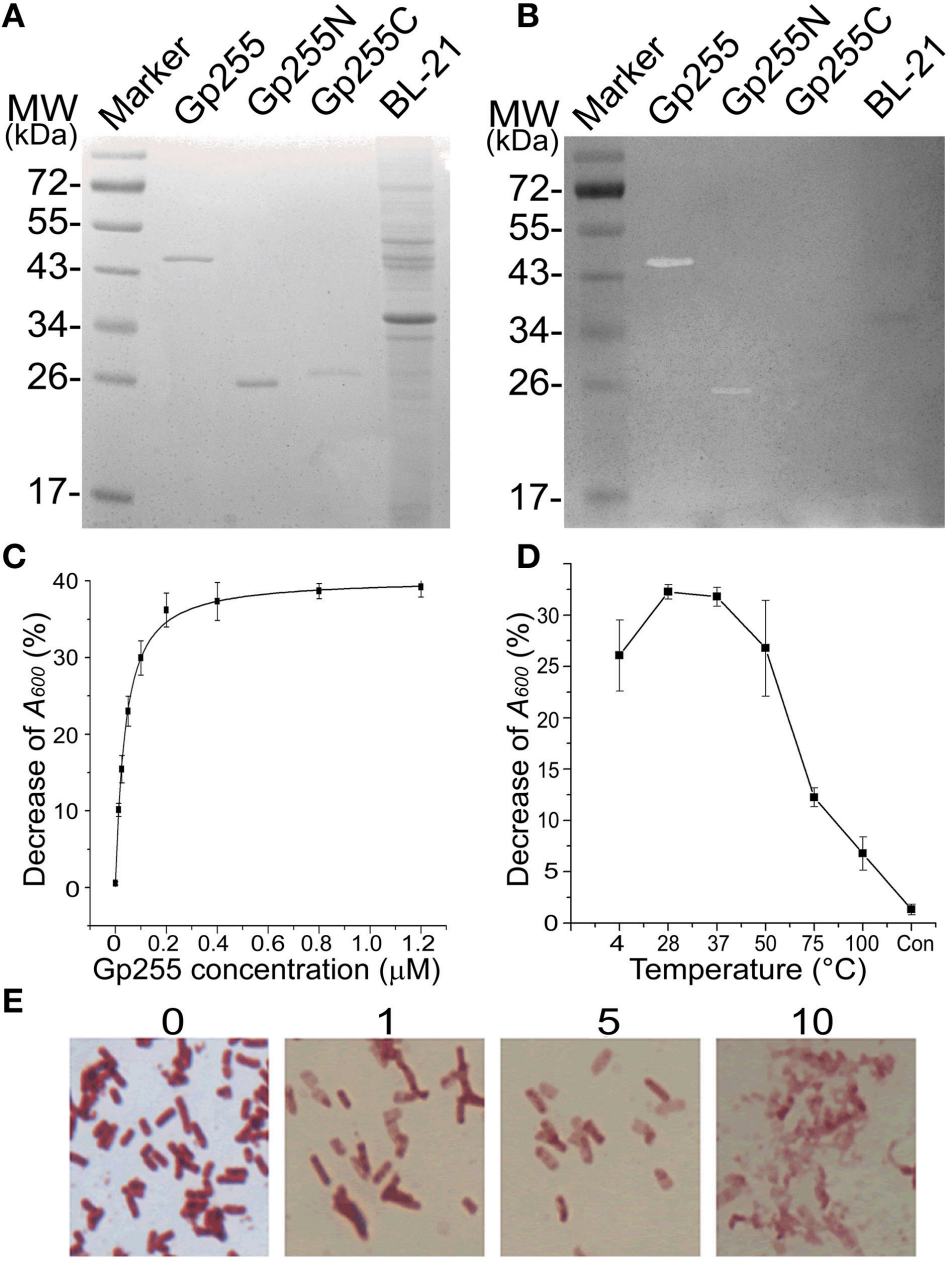
**Characterization of protein Gp255**. **(A)** SDS-PAGE analysis of purified Gp255, Gp255N, Gp255C, and *E. coli* strain BL-21. **(B)** In-gel lytic activities of proteins Gp255, Gp255N, Gp255C, and *E. coli* strain BL-21. The purified proteins were mixed with loading buffer for use. **(C)** The influence of protein concentration on the lytic curve of Gp255. Gp255 was used at different concentration and the test was carried out at 37°C. **(D)** Temperature tolerance of Gp255. Gp255 at a concentration of 10 μM was treated at different temperatures for 10 min and the treated proteins were used for lytic activity evaluation at a final concentration of 0.05 μM. The cells treated with PBS solution was used as control (indicated as “Con”). **(E)** Lytic activity of Gp255 against *B. pumilus* strain GR8. The strain GR8 was observed by optical microscope at 0, 1, 2, and 10 min after treatment and the time of observation is indicated. The purified Gp255 was used with a final concentration of 0.05 μM and the tests were carried out at 37°C.

**Figure 4:**
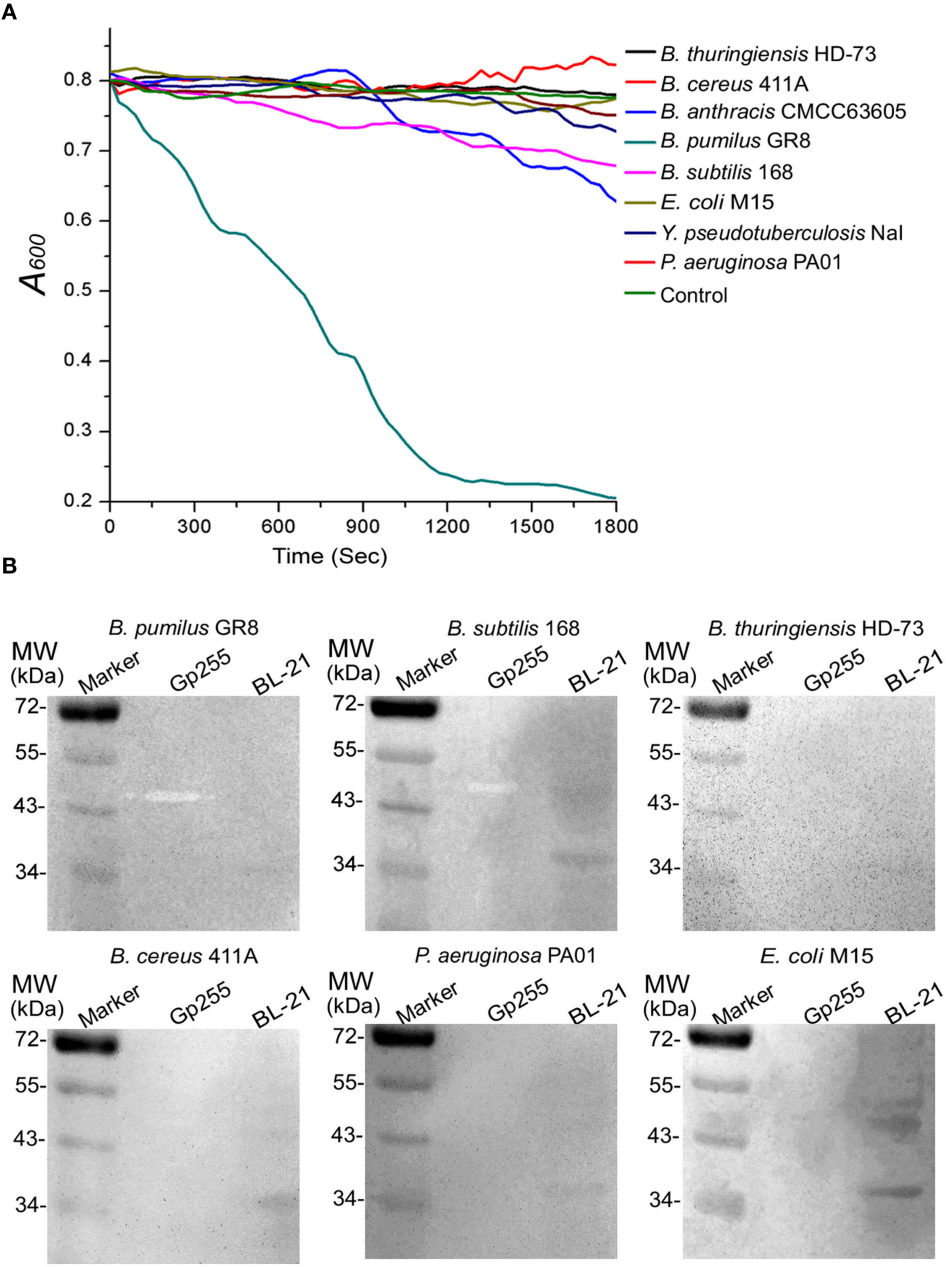
**Lytic specificity of Gp255. (A)** Lytic activity of Gp255 against eight tested strains. The suspension of exponential-growth GR8 without adding Gp255 was used as control. For the Gram-negative strain, EDTA was added into the reaction buffer at a concentration of 1 mM. **(B)** In-gel lytic activity assay of Gp255. The crude cell extracts of six strains were used.

Glycoside hydrolase protein Gp255 encoded by gene *gp*255 in the phage vB_BpuM_BpSp genome was expressed and its function was analyzed. G255 could lyze the tested cell extracts of strain GR8 and N-termini of protein Gp255 was proved to be the catalytic domain of the protein (Figures 3A,B). Purified protein Gp255 exhibited high lytic activity against the host strain *B. pumilus* GR8 at a low concentration of 0.0125 μM (Figure 3C). Temperature tolerance assay of Gp255 indicated that it has high temperature tolerance and can maintain its lytic activity even after exposure to 100°C for 10 min (Figure 3D). Gp255 caused the cell lysis of strain GR8 by a similar catalytic mechanism similar to that of phage endolysin by degrading the cell wall, thus causing further the damage of the bacterial cells (Figure 3E). Gp255 showed high lytic specificity to the tested strain GR8 and weak lytic activities to the tested strains of *B. subtilis* and *B. anthracis*, and no lytic activities against the tested strains of *B. thuringiensis, B. cereus, E. coli, Y. pseudotuberculosis*, and *P. aeruginosa* (Figure 4).

### Peptidoglycan-hydrolyzing activities of vB_BpuM_BpSp structural proteome and Gp255

**Figure 5:**
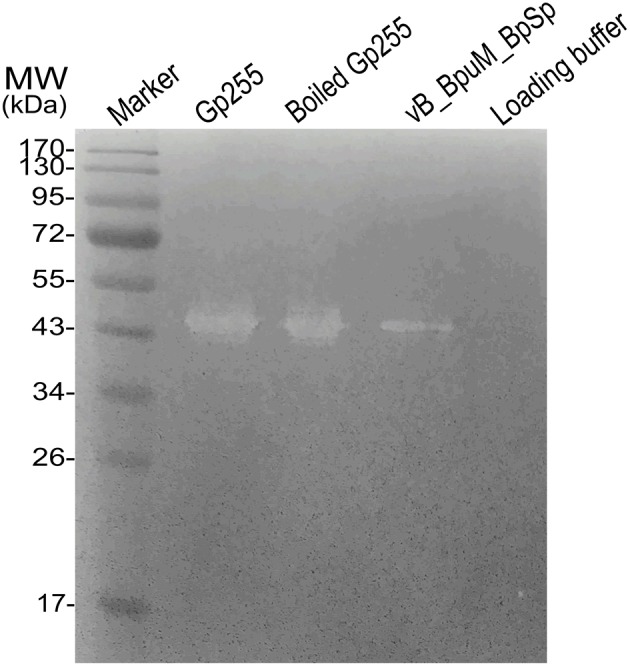
**Analysis of peptidoglycan-hydrolyzing activities of phage vB_BpuM_BpSp**. The purified phage vB_BpuM_BpSp was mixed with loading buffer and boiled at 100°C for 10 min before separating them by SDS-PAGE. The purified Gp255 was mixed with loading buffer. After that, the unboiled (indicated as “Gp255”) and boiled (indicated as “Boiled Gp255”) proteins were separated by SDS-PAGE. The sterilized crude cell extracts of GR8 was added into the gel for lytic activity assay. The lane loaded with 1 × Loading buffer were used as control.

To analyze the existence and activity of Gp255 in the phage structural proteome, the phage proteome and purified protein Gp255 was separated by SDS-PAGE and the peptidoglycan-hydrolyzing activities were tested *in situ* against the crude cell extract of strain GR8. The results indicate that both the boiled and unboiled protein Gp255 show high lytic activities against the bacterial cell extracts in the gel (Figure 5). Though huge reduction of lytic activity after exposure to 100°C was observed in Figure 3D, similar activities of Gp255 and boiled Gp255 were observed in the gel, which might be because the lytic activity of the boiled Gp255 was recovered after treatment of renaturation buffer. A degradation zone with almost the same molecular weight as that of Gp255 lane was observed in the lane of phage vB_BpuM_BpSp, indicating that Gp255 is a component of the phage vB_BpuM_BpSp and the protein in phage virion still displays peptidoglycan hydrolase activity.

### Interaction of Gp255 and phage baseplate protein Gp287

**Figure 6:**
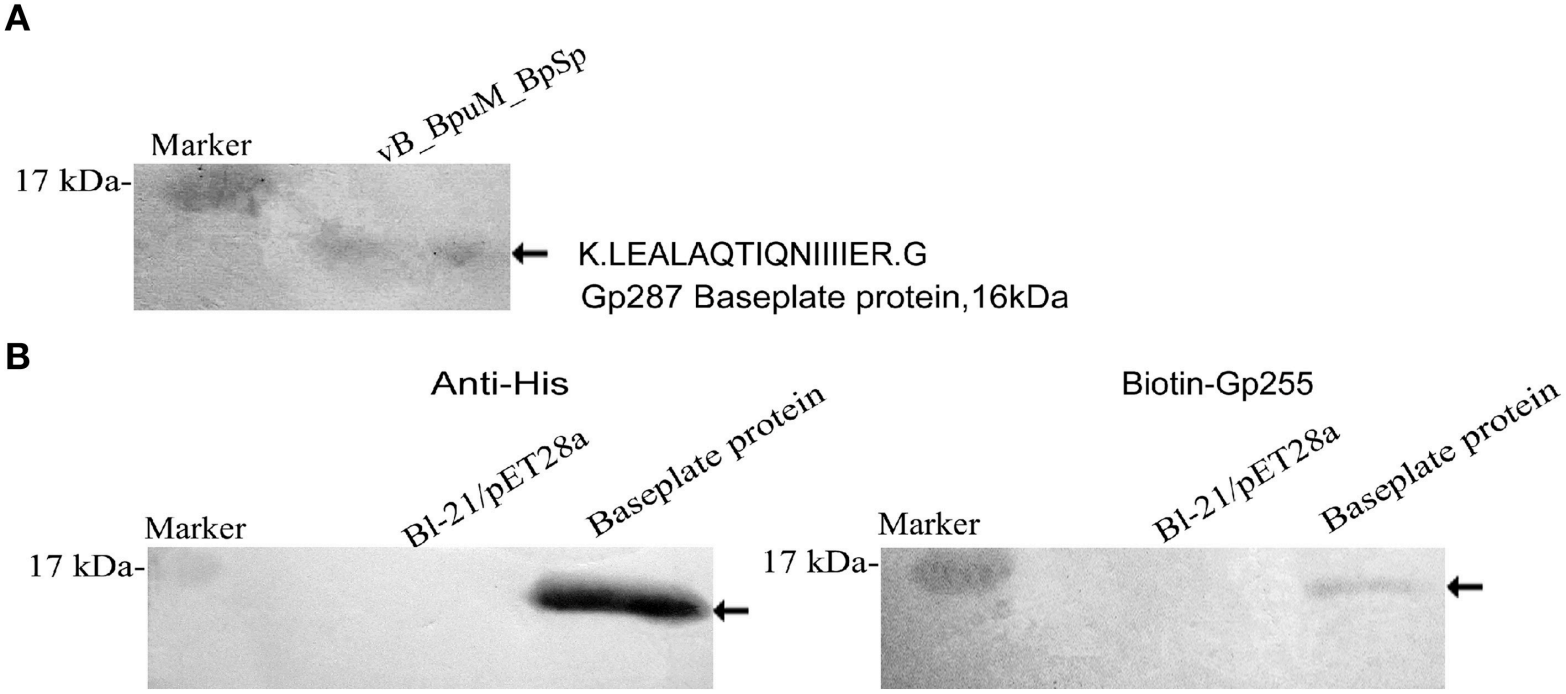
**Identification of the phage structural protein that interacted with Gp255**. **(A)** Identification of the interacting protein of Gp255 in phage vB_BpuM_BpSp structural proteome. The virions of phage vB_BpuM_BpSp were separated by SDS-PAGE and biotin-labeled Gp255 was used for Far-western blotting analysis. The detected protein band was identified by MALDI-TOF/-TOF MS. The identified peptide and corresponding protein encoded by phage vB_BpuM_BpSp are indicated. **(B)** Verification of the interaction between Gp255 and Gp287. Purified Gp287 were separated by 15% SDS-PAGE. *E. coli* strain BL-21/pET28a was used as control. Western blotting was carried by using the antibody of 6 × His-tag and the biotin-labeled Gp255, respectively. The detected hybridization bands are indicated.

Purified Gp255 was labeled with biotin and a Far western blotting analysis of Gp255 was carried out. The results indicate that labeled Gp255 possibly interacts with a unique protein with a molecular weight of 16 kDa in the structural proteome of phage vB_BpuM_BpSp, and the protein was identified as the phage baseplate protein (Gp287) by MALDI-TOF-TOF MS (Figure 6A). Protein Gp287 was expressed and the interaction between Gp255 and Gp287 was verified by Far western blotting analysis, suggesting Gp255 might indeed interact with Gp287 (Figure 6B). According to previous reports, the polysaccharide lytic enzyme domain acts as a part of the phage tail fiber or tail spike proteins, which interacted with the baseplate of the phage (Yap and Rossmann, 2014). Our result showed that Gp255 could also interact with the baseplate substructure. SDS-PAGE analysis of the structural proteome of phage vB_BpuM_BpSp showed that Gp255 is highly abundance (Figure 1), suggesting that the baseplate substructure containing Gp255 might be a major substructure of phage vB_BpuM_BpSp.

## Discussion

The newly isolated Jumbo phages have often been found to exhibit low similarity to existing biological entities and show numerous uncharted features. The genomic annotation of Jumbo phage genome is often disappointing because most of the predicted proteins, including the structural proteins, fail to make good hits to the sequences in the databases. The genomic annotation of Jumbo phage vB_BpuMP_BpSp only revealed eight predicted phage structural proteins, and the genes encoding other phage structural proteins remain unknown. Structural proteomic analysis combined with bioinformatic analysis of phage vB_BpuM_BpSp identified 22 phage-coding structural proteins, including 14 proteins with unknown function, a glycoside hydrolase, a Holliday junction resolvase, a RNA polymerase (RNAP) β subunit, and five other annotated structural proteins. Among the 14 proteins of unknown function, the candidates for the curly phage tail fiber protein and the major capsid protein were predicted by bioinformatic analysis. To our knowledge, glycoside hydrolase, Holliday junction resolvase, and RNA polymerase (RNAP) β subunit are rarely found in phage virions. Our findings of new structural proteins of phage vB_BpuM_BpSp suggests that structural proteomic analysis of phage virions is a promising approach for the identification of unknown phage structural components.

Besides the identified structural proteins encoded by phage vB_BpuM_BpSp genome, a portal protein encoded by the host strain genome was found. Either the host-coding portal protein, instead the phage-coding portal protein, is hijacked by the phage vB_BpuM_BpSp to assemble progeny phage particle, or the portal protein is packaged into the phage virion by mistake in some non-function way. Besides the identified portal protein, the genome of the host strain GR8 also encode 15 more phage portal protein homologs, which were not identified by proteomic analysis of phage virions, further supporting the speculation that the identified portal protein might be a functional component hijacked by phage vB_BpuM_BpSp from the host strain.

The RNA polymerase β subunit was identified as one of the phage vB_BpuM_BpSp structural proteins. It was also found in the virion proteome of phage 201ϕ2-1, and possibly in that of *B. subtilis* phage PBS2 as well, and is thought to be phage virion-associated RNAP, which might be injected into the host cell during phage infection to facilitate the synthesis of phage proteins at the early stage after infection, including the genome replication-associated proteins and phage specific proteins, before the expression of other phage RNAPs (Clark et al., 1974; Thomas et al., 2008). Another identified structural protein, the Holliday junction resolvase, binds and cleaves the junction in the phage genome to further facilitate the replication of phage genome, the packaging of circular genome during phage assembly, and the release of condensed genome during phage infection by localizing at portal of the phage head (Golz and Kemper, 1999; Dixit et al., 2011; Green et al., 2013). The Holliday junction resolvase in phage virion might be the remnant of the phage genome package device or the facilitating mechanism for the translocation of large, condensed phage genome to host cell during infection. Phage vB_BpuM_BpSp has a large genome, and the packaging and release of the phage genome is an energy consuming process. The structural RNA polymerase β subunit and Holliday junction resolvase identified in the vB_BpuM_BpSp proteome might be components of an adaption mechanism to overcome these problems. Further confirmation of these findings and functional analysis of these two proteins would shed more light on their roles in phage virions.

Phages recognize host cell by the “walk” of the tail fiber on the cell surface (Hu et al., 2015). The reversible binding of phage tail fibers to bacterial cell wall leads the phage to the optimal site for irreversible absorption and subsequent infection. Jumbo phage vB_BpuM_BpSp has a fragile head, and the tail sheath substructure is easily separated. The curly tail fiber of vB_BpSp_BpSp is longer than the tail fiber of myovirus with smaller genome (Hu et al., 2015) and it swing more easily. The long, flexible tail fiber might lead the phage vB_BpuM_BpSp to optimal infection site quickly and reduce the damage of phage virion during recognition. Similar helical tail fibers have only been observed in the *B. thuringiensis* phage 0305ϕ8-36 (Serwer et al., 2007), *B. subtilis* phage AR9 (Belyaeva and Azizbeky, 1968), *B. subtilis* phage PBS1 (Eiserlin, 1967), *B. cereus* phage Bace-11 (Ackermann et al., 1995), and *Salmonella* phage c (Schade et al., 1967). Among these six known phages, the hosts of five are strains of *Bacillus* and only phage 0305ϕ8-36 is a Jumbo phage. Proteomic analysis and comparative genomic analysis revealed that Gp133 of phage vB_BpuM_BpSp is a phage structural protein with similarity to the predicted curly tail fiber protein ORF129 of phage 0305ϕ8-36, suggesting that Gp133 might be a component of the curly tail fiber. Further functional analysis of the protein will provide more understanding to the function of curly tail fibers in *Bacillus* phage virion.

The Jumbo phages have huge phage virions and large genomes, which results in high energy consumption during infection. Several genes in the vB_BpuM_BpSp genome exhibit potential functions for facilitating phage infection, such as the genes of glycoside hydrolase and Holliday junction resolvase. The peptidoglycan lytic enzyme domain has been found to be a part of the phage tail fiber and tail spike with function of benefiting infection (Boulanger et al., 2008; Thompson et al., 2010). However, no individual protein with identified glycoside hydrolase activity has been found in phage virion. This study identified the first functional individual phage glycoside hydrolase with a structural function. This glycoside hydrolase, Gp255, was found to interact with the baseplate protein of the phage, but not with the tail spike protein or the tail fiber protein. The infection of myovirus undergoes a series of structural and conformational changes and the changes of baseplate conformation leads to the release of short tail fiber, which interacts with the cell wall (Hu et al., 2015). During this process, the glycoside hydrolase of phage vB_BpuM_BpSp might be released and it may catalyze the pore formation for phage genomic DNA injection. This mechanism might reduce the lytic time of the glycoside hydrolase to the cell wall, and the damage of the glycoside hydrolase to cell wall, and is helpful for maintaining conducive intracellular environment for phage propagation.

## Author contributions

MG and YY conceived and designed the work; YY performed the experiment and analyzed the data. YY and MG draft the manuscript; MG revised the manuscript.

## Funding

This study was supported by the National Natural Science Foundation of China (No. 31500155, 31170123) and the projects of Chinese Academy of Sciences (KSZD-EW-Z-021-2-2). The funders had no role in study design, data collection and interpretation, or the decision to submit the work for publication.

### Conflict of interest statement

The authors declare that the research was conducted in the absence of any commercial or financial relationships that could be construed as a potential conflict of interest.
